# Supplementary material for: Therapeutic Efficacy of Helianthemum lippii Extract and Silver Nanoparticles Synthesized from the Extract against Cadmium-Induced Renal Nephrotoxicity in Wistar Rats
Source: Pharmaceuticals (Basel). 2024 Jul 25;17(8):982. doi: 10.3390/ph17080982 (PMC11357364; doi:10.3390/ph17080982)
Supplement: Supplementary file 1 [file pharmaceuticals-17-00982-s001.zip › pharmaceuticals-3108282-supplementary.pdf]

pharmaceuticals

Supplementary information

Table S1. Baseline body weight, body weight changes between baseline and the study end, and relative kidney weight at the study end in the seven rat groups. Group 1 (Control): not treated/not exposed. Group 2 (Cd): addition of CdCl2 (50 mg/kg body weight/day) in drinking water for 35 days. Group 3 (Hl): H. lippii (100 mg/kg, by gavage for 35 days). Group 4 (Cd + Hl): CdCl2 exposure (like in Group 2) followed by H. lippii (100 mg/kg, body weight/day by gavage) for 15 days. Group 5 (Ag): Ag NPs (100 µg/kg body weight/day, intraperitoneal injection) for 35 days. Group 6 (Cd + Ag): CdCl2 exposure (like in Group 2) followed by Ag NPs (0.1 mg/kg body weight/day by intraperitoneal injection) for 15 days. Group 7 (Cd+Hl+Ag): CdCl2 exposure (like in Group II) followed by H. lippii (100 mg/kg body weight/day by gavage) and Ag NPs (0.1 mg/kg body weight/day by intraperitoneal injection) for 15 days. ***$p < 0.001$ vs Group 1; a $p < 0.05$, b $p < 0.01$, C $p < 0.001$: vs Group 2 (n = 5 rats/group).

Rat groups	Group 1 (Control)	Group 2 (Cd)	Group 3 (*Hl*)	Group 4 (Cd+*Hl*)	Group 5 (Ag)	Group 6 (Cd + Ag)	Group 7 (Cd+*Hl*+Ag)
Parameters	Drinking water	$CdCl_2$ in drinking water for 35 days	*H. lippii* by gavage 100 mg/kg for 35 days	*H. lippii* after exposure to $CdCl_2$	Ag NPs by intraperitoneal injection for 15 days	Ag NPs after exposure to $CdCl_2$	*H. lippii* and Ag NPs after exposure to $CdCl_2$
Initial body weight (g)	282±14.7	204.2±21.9	244.8±2.69	247.2±1.1	231.8±15.7	233.8±8.95	234.0±13.0
Body weight gain (g/d)	0.072±0.02	-0.18±0.06***	0.119±0.09	-0.05±0.2[a]	0.042±0.01	0.07±0.009[c]	0.02±0.03[a]
Relative kidney weight (g/100 g body weight)	0.46±0.04	0.55±0.02***	0.43±0.03	0.48±0.01[a]	0.42±0.02	0.48±0.02[b]	0.47±0.01[a]

Table S2. Serum biochemical markers in the different rat groups at the study end. Group 1 (Control): not treated/not exposed. Group 2 (Cd): addition of $CdCl_2$ (50 mg/kg body weight/day) in drinking water for 35 days. Group 3 (*Hl*): *H. lippii* (100 mg/kg body weight/day, by gavage for 35 days). Group 4 (Cd + *Hl*): $CdCl_2$ exposure (like in Group 2) followed by *H. lippii* (100 mg/kg body weight/day by gavage) for 15 days. Group 5 (Ag): Ag NPs (100 µg/kg body weight/day, intraperitoneal injection) for 35 days. Group 6 (Cd + Ag): $CdCl_2$ exposure (like in Group 2) followed by Ag NPs (0.1 mg/kg body weight/day by intraperitoneal injection) for 15 days. Group 7 (Cd+Hl+Ag): $CdCl_2$ exposure (like in Group II) followed by *H. lippii* (100 mg/kg body weight/day by gavage) and Ag NPs (0.1 mg/kg body weight/day by intraperitoneal injection) for 15 days. **p < 0.01, ***p < 0.001 vs Group 1; a p < 0.05, C p < 0.001: vs Group 2 (n = 5 rats/group).

Rat Groups	Serum triglycerides (g/l)	Serum cholesterol (mg/l)	Serum glucose (g/I)	Serum albumin (g/l)
Group 1 (Control)	0.39±0.02	0.62± 0.001	0.86±0.04	30± 0.6
Group 2 (Cd)	0.79±0.01***	0.78± 0.02**	1.13± 0.09**	29.5± 0.28**
Group 3 (*Hl*)	0.65±0.01	0.66± 0.01	0.79±0.05	31± 0.31
Group 4 (Cd+*Hl*)	0.63±0.01[c]	0.75± 0.01	0.82± 0.10[c]	31.33± 0.44[c]
Group 5(Ag)	0.64±0.04	0.62± 0.04	1.03± 0.09	31± 0.56
Group 6 (Cd + Ag)	0.59±0.05	0.63± 0.03[a]	1.08± 0.009[c]	29.66± 0.4[c]
Group 7 (Cd+*Hl*+Ag)	0.40±0.01[c]	0.75± 0.01[a]	0.88± 0.06[c]	30.75± 0.3[c]

Table S3. Kidney function markers in the seven rat groups. Group 1 (Control): not treated/not exposed. Group 2 (Cd): addition of $CdCl_2$ (50 mg/kg body weight/day) in drinking water for 35 days. Group 3 (*Hl*): *H. lippii* (100 mg/kg body weight/day, by gavage for 35 days). Group 4 (Cd + *Hl*): $CdCl_2$ exposure (like in Group 2) followed by *H. lippii* (100 mg/kg body weight/day by gavage) for 15 days. Group 5 (Ag): Ag NPs (100 µg/kg body weight/day, intraperitoneal injection) for 35 days. Group 6 (Cd + Ag): $CdCl_2$ exposure (like in Group 2) followed by Ag NPs (0.1 mg/kg body weight/day by intraperitoneal injection) for 15 days. Group 7 (Cd+Hl+Ag): $CdCl_2$ exposure (like in Group II) followed by *H. lippii* (100 mg/kg body weight/day by gavage) and Ag NPs (0.1 mg/kg body weight/day by intraperitoneal injection) for 15 days. *$p < 0.05$, ***$p < 0.001$ vs Group 1; a $p < 0.05$, b $p < 0.01$, C $p < 0.001$ vs Group 2 (n = 5 rats/group).

Rat groups	Serum urea (g/l)	Serum creatinine (mg/l)	Serum uric acid (mg/l)	Urea/creatinine ratio
Group 1 (Control)	0.50±0.03	4.78±0.08	11.5±0.56	94.04±8.36
Group 2 (Cd)	0.58±0.02*	5.36±0.001***	20.00±0.001*	108.0±11.2*
Group 3 (*Hl*)	0.45±0.022	4.81±0.05	11.33±1.45	94.87±6.72
Group 4 (Cd+*Hl*)	0.47±0.028[b]	4.25±0.001[c]	16.25±1.50[a]	110.9±10.0
**Group 5(Ag)**	0.46±0.025	4.010±0.0001	11.67±1.28**	104.74±8.60
**Group 6 (Cd + Ag)**	0.47±0.01[c]	5.11±0.08[a]	12.5±0.77[c]	95.25± 6.46[a]
Group 7 (Cd+*Hl*+Ag)	0.48±0.02[a]	4.81±0.08[c]	11.75±0.55[c]	95.1±11.4[a]

Table S4. Kidney oxidative stress markers in the seven rat groups. Group 1 (Control): not treated/not exposed. Group 2 (Cd): addition of $CdCl_2$ (50 mg/kg body weight/day) in drinking water for 35 days. Group 3 (*Hl*): *H. lippii* (100 mg/kg body weight/day, by gavage for 35 days). Group 4 (Cd + *Hl*): $CdCl_2$ exposure (like in Group 2) followed by *H. lippii* (100 mg/kg body weight/day by gavage) for 15 days. Group 5 (Ag): Ag NPs (100 µg/kg body weight/day, intraperitoneal injection) for 35 days. Group 6 (Cd + Ag): $CdCl_2$ exposure (like in Group 2) followed by Ag NPs (0.1 mg/kg body weight/day by intraperitoneal injection) for 15 days. Group 7 (Cd+Hl+Ag): $CdCl_2$ exposure (like in Group II) followed by *H. lippii* (100 mg/kg body weight/day by gavage) and Ag NPs (0.1 mg/kg body weight/day by intraperitoneal injection) for 15 days. ***$p < 0.001$ vs Group 1; a $p < 0.05$, b $p < 0.01$, C $p < 0.001$ vs Group 2 (n = 5 rats/group).

Rat groups	MDA (nmol/mg protein)	SOD (U/mg protein)	GSH (nmol/mg tissue)	Catalase (U/g tissue)
Group 1 (Control)	0.403±0.021	0.26±0.009	0.00023±0.001	0.04373±0.008
Group 2 (Cd)	0.9061±0.0592***	0.239±0.00001	0.00022±0.000001	0.01931±0.0072***
Group 3 (*Hl*)	0.418±0.0647	0.242±0.006	0.00025±0.0001	0.044±0.0020
Group 4 (Cd+*Hl*)	0.7469±0.0545[a]	0.269±0.0109[a]	0.00025±0.00001[c]	0.04236±0.0061[b]
**Group 5 (Ag)**	0.577±0.0389	0.2819±0.0142	0.00025±0.00001	0.0397±0.0009
**Group 6 (Cd + Ag)**	0.4919±0.0594[c]	0.272±0.00001[c]	0.00024±0.00001[a]	0.02835±0.00202[c]
Group 7 (Cd+*Hl*+Ag)	0.5274±0.0382[c]	0.257±0.0032[c]	0.00024±0.00001[b]	0.0458±0.0108[b]

Table S5. Semi-quantitative results of the histological analysis of the kidney architecture in the different rat groups: Group 1 (Control): not treated/not exposed. Group 2 (Cd): addition of $CdCl_2$ (50 mg/kg body weight/day) in drinking water for 35 days. Group 3 (*Hl*): *H. lippii* (100 mg/kg body weight/day, by gavage for 35 days). Group 4 (Cd + *Hl*): $CdCl_2$ exposure (like in Group 2) followed by *H. lippii* (100 mg/kg body weight/day by gavage) for 15 days. Group 5 (Ag): Ag NPs (100 µg/kg body weight/day, intraperitoneal injection) for 35 days. Group 6 (Cd + Ag): $CdCl_2$ exposure (like in Group 2) followed by Ag NPs (0.1 mg/kg body weight/day by intraperitoneal injection) for 15 days. Group 7 (Cd+Hl+Ag): $CdCl_2$ exposure (like in Group II) followed by *H. lippii* (100 mg/kg body weight/day by gavage) and Ag NPs (0.1 mg/kg body weight/day by intraperitoneal injection) for 15 days (n = 5 rats/group).

Rat groups	Group 1	Group 2	Group 3	Group 4	Group 5	Group 6	Group 7
Parameters	Control	Cd	*Hl*	Cd + *Hl*	Ag	Cd +Ag	Cd +Ag+*Hl*
Inflammatory infiltration	-	+++	-	-	-	-	-
Tubular dilation	-	+++	-	+	-	-	-
Destroyed glomeruli	-	+++	-	-	-	-	-
